# Two Novel AP2/EREBP Transcription Factor Genes *TaPARG* Have Pleiotropic Functions on Plant Architecture and Yield-Related Traits in Common Wheat

**DOI:** 10.3389/fpls.2016.01191

**Published:** 2016-08-09

**Authors:** Bo Li, Qiaoru Li, Xinguo Mao, Ang Li, Jingyi Wang, Xiaoping Chang, Chenyang Hao, Xueyong Zhang, Ruilian Jing

**Affiliations:** National Key Facility for Crop Gene Resources and Genetic Improvement, Institute of Crop Science, Chinese Academy of Agricultural SciencesBeijing, China

**Keywords:** wheat, AP2/EREBP transcription factor, *TaPARG*, plant architecture, yield

## Abstract

AP2/EREBPs play significant roles in plant growth and development. A novel, pleiotropic *TaPARG* (*PLANT ARCHITECTURE-RELATED GENE*), a member of the AP2/EREBP transcription factor gene family, and its flanking sequences were isolated in wheat (*Triticum aestivum* L.). Two *TaPARG* genes were identified and named as *TaPARG-2A* and *TaPARG-2D*. Their amino acid sequences were highly similar especially in the functional domains. *TaPARG-2A* on chromosome 2A was flanked by markers *Xwmc63* and *Xgwm372*. *TaPARG-2D* was mapped to chromosome 2D. Subcellular localization revealed that TaPARG-2D was localized in the nucleus. The results of tissue expression pattern, overexpression in rice, association analysis and distinct population verification jointly revealed that *TaPARG* functions during the entire growth cycle of wheat. Its functions include regulation of plant architecture-related and yield-related traits. Association analysis, geographic distribution and allelic frequencies suggested that favored haplotypes *Hap-2A-2* and *Hap-2A-3* were selected in Chinese wheat breeding programs. Both favored haplotypes might be caused by a single amino acid substitution (His/Tyr). These results suggest that *TaPARG* is a regulatory factor in plant growth and development, and that the favored alleles might be useful for improving plant architecture and grain yield of wheat.

## Introduction

AP2/EREBPs (ethylene-responsive element-binding proteins) belong to a superfamily of plant-specific transcription factors characterized by presence of an AP2 DNA-binding domain of 60 amino acids (Weigel, 1995; Okamuro et al., 1997). The highly conserved amino acid residues of AP2 domains were also called the GCC-box binding domain (GBD), because of specific binding to the GCC-box (Ohme-Takagi and Shinshi, 1995; Allen et al., 1998). The AP2/EREBP superfamily is divided into two subfamilies based on the number of AP2 domains (Riechmann and Meyerowitz, 1998). EREBPs containing a single AP2 domain are involved in regulatory networks of response to hormones, pathogen attack, and environmental signals involving DREBs (dehydration responsive element binding proteins) and ERFs (ethylene responsive factors) (Xu et al., 2011; Licausi et al., 2013; Jisha et al., 2015). EREBPs with two tandom AP2 domains (known as repeat 1 and repeat 2) have roles in the regulation of plant development; the AP2 subfamily is a good example (Zhao et al., 2006; Wang et al., 2014; Kuluev et al., 2015).

Previous studies demonstrated that members of the AP2 subfamily have roles in controlling flower and seed developmental processes. *APETALA2* (*AP2*), with an important role in flowering and seed development in *Arabidopsis* encodes a putative transcription factor distinguished by a novel DNA binding motif referred to as the AP2 domain (Okamuro et al., 1997). In *Arabidopsis AP2* is also involved in determination of seed size, seed weight, and accumulation of seed oil and protein (Jofuku et al., 2005). The *AINTEGUMENTA* (*ANT*) gene regulates flower development and is essential for ovule formation (Klucher et al., 1996). BABY BOOM (BBM) triggers conversion from vegetative to embryonic growth during embryogenesis (Boutilier et al., 2002). The *PLETHORA1* (*PLT1*) and *PLETHORA2* (*PLT2*) genes are key effectors for establishment of the stem cell niche during embryonic pattern formation in *Arabidopsis* (Aida et al., 2004). The *AINTEGUMENTA*-*like* (*AIL*) genes are expressed in young tissues and may specify meristematic or divison-competent states (Nole-Wilson et al., 2005). *AINTEGUMENTA-like* gene *NtANTL*, a putative ortholog of *AtANT*, controls cell division and expansion in controlling organ growth in tobacco (Kuluev et al., 2015). Extensive research has been conducted on the AP2/EREBP family of transcription factors in wheat, but has mainly focused on the DREB and ERF subfamilies because their overexpression enhances tolerance to abiotic and biotic stresses (Xu et al., 2011); however, some AP2 genes have been studied and showed their functions in plant growth and development. For example, gene *Q* which has a high degree of similarity to members of AP2 family has pleiotropically influences on many domestication-related traits, such as glume shape and tenacity, rachis fragility, spike length, plant height (PH), and spike emergence time (Simons et al., 2006). The engineering of cleistogamous wheat requires the presence of a functional *TaAP2* modification at each of the three homoeologs (Ning et al., 2013).

In this study, the novel, pleiotropic gene, designated *TaPARG* (*PLANT ARCHITECTURE-RELATED GENE*), was isolated and characterized in wheat. Our findings suggest that *TaPARGs* play a key role in growth and development, including regulation of plant architecture-related traits such as PH, tiller number, and yield-related traits, including fertility and 1,000 kernel weight (TKW). The coding region of *TaPARG-2A* was analyzed, and sequence polymorphisms among common wheat cultivars were identified. Finally, functional markers for various haplotypes were developed, and favored haplotypes were identified by association analysis of two germplasm populations. The favored haplotypes *Hap-2A-2* and *Hap-2A-3* were selected in Chinese wheat breeding programs. Both favored haplotypes probably arose by a single amino acid change (His/Tyr). Our overall results suggest that *TaPARGs* are pleiotropic genes involved in growth and development, and that two functional markers developed for *TaPARG-2A* might be helpful for marker-assisted selection of ideal plant architecture and high-yielding wheat genotypes.

## Materials and Methods

### Plant Materials and Measurement of Agronomic Traits

Hanxuan 10 is a drought-tolerant cultivar released in 1966 and still grown in arid and barren areas, and also one of the strongly drought-tolerant accessions selected from more than 20,000 wheat germplasm resources. Therefore, Hanxuan 10 is used as the plant material for cloning genes *TaPARG-2A* and *TaPARG-2D*, and to analysis the polymorphism of *TaPARGs* and its relation with the agronomic traits under diverse water conditions. Lumai 14 is a high-yielding cultivar adapted to abundant water and fertile conditions, and is widely grown during the 1990s in northern China. Using Hanxuan 10 and Lumai 14 as parents, we developed a doubled haploid (DH) population and mapped QTLs for a list of important agronomic traits (Yang et al., 2007; Wang et al., 2010; Wu et al., 2011, 2012). Hanxuan 10 was used for gene cloning and expression analysis. Thirty-four diverse cultivars (Supplementary Table S1) were used to identify sequence polymorphism of *TaPARG*. A set of nullisomic-tetrasomic and ditelosomic lines of Chinese Spring were used for chromosome location of the gene. A doubled haploid (DH) population derived from the cross Hanxuan 10 × Lumai 14 (Yang et al., 2007) was employed for genetic mapping. Kitaake, a short life cycle rice cultivar, was used for genetic transformation.

Three common wheat germplasm populations were used in association studies of *TaPARG* haplotypes and phenotypic traits. The Population 1 (262 accessions) was firstly used; the accessions were mainly from the Northern Winter Wheat and Yellow and Huai River Valleys Facultative Wheat Zones (Zhang et al., 2013). Population 2 (157 landraces) and Population 3 (348 modern cultivars) including genotypes from all 10 Chinese wheat production zones were used to verify the results of the initial association analysis, allelic frequencies and geographic distribution analysis. Population 2 entries mainly came from the Chinese wheat mini-core collection, which represents more than 70% of the total genetic diversity of the Chinese wheat germplasm collection, and Population 3 entries were from the Chinese wheat core collection (Hao et al., 2008, 2011).

Population 1 was planted at Shunyi (40°23′N; 116°56′E) and Changping (40°13′N; 116°13′E), Beijing, over 3 years (2010–2012) for measurement of agronomic traits, including PH, peduncle length (PL), top 2nd internode length (TSL), effective tiller number per plant (ETN), and TKW. Two water regimes, rain-fed (drought stressed, DS) and well-watered (WW) were applied at each site. The Population 3 was planted in three environments in 2002 and 2005 at Luoyang (34°61′N; 112°45′E) in Henan province, and at Shunyi, Beijing, in 2010. Each plot contained four 2 m rows spaced 30 cm apart, with 40 plants in each row. The PH was measured in the field. At mature stage, five plants were randomly sampled from the middle position of each plot to measure ETN, and the main tillers were used for PL and TSL measurement.

Transgenic and wild type (WT) rice accessions were planted at the Institute of Crop Science Experimental Station in Beijing (39°48′ N, 116°28′ E) in an area dedicated for transgenic plants. The field trial was managed in randomized complete blocks with three replicates. Each plot contained three 1.5 m rows spaced 15 cm apart, with 15 plants in each row. At mature stage, 10 plants were randomly sampled from the middle position of each plot to measure tiller number and 1,000 kernel weight, the main panicles were used for measuring plant height and seed setting rate.

### Cloning *TaPARGs* in Wheat

To obtain coding and flanking sequences of *TaPARGs*, the conserved sequence of the AP2 domain was used to blast against the draft genomes of the wheat putative diploid progenitors *Triticum uratrtu* (A genome; Ling et al., 2013) and *Aegilops tauschii* (D genome; Jia et al., 2013). Three pairs of primers were designed according to the assembled *TaPARG* sequence. They was a common primer pair, F1 (5′-GCATTG ACCATTAGCTGGTCT-3′) and R1 (5′-TCCGACCGAGTGCT CATT-3′) to amplify simultaneously the full-length cDNA of *TaPARG-2A* and *TaPARG-2D* by PCR; a A genome-specific primer pair (F2, 5′-CGCAAAAACACACTTGCTCA-3′ and R2, 5′-TCCGACCGAGTGCTCATT-3′) to amplify *TaPARG-2A*, including the 5′ and 3′ flanking regions; and a D genome-specific primer pair (F3, 5′-TACTTCTTTAGGGGTATCGCC-3′, R3, 5′-CGCTCTGGTCACAATGACAC-3′) to amplify *TaPARG-2D*. Gene structure of *TaPARG* was determined using DNASTAR Lasergene 7.10 (DNASTAR, Inc., Madison, WI, USA).

### Chromosome Location

Fifty-five nulli-tetrasomic and ditelosomic lines of Chinese Spring were used for chromosome location of *TaPARG-2D*. *TaPARG-2A* was mapped using the DH population (Hanxuan 10 × Lumai 14) with the MAPMAKER/EXP 3.0 (Lander and Botstein, 1989).

### Subcelluar Localization of *TaPARG* Protein

TaPARG-2D was fused upstream of GFP in the pCAMBIA1300 vector under control of the CaMV 35S promoter. Primers 5′-CCC AAGCTTATGACCAACAACAACGGCA-3′ (*Hin*dIII site underlined) and 5′-GGGGTACCTGCGTCGCTCCACGC-3′ (*Kpn*I site underlined) were used for sub-cloning. The constructs were transferred into wheat mesophyll protoplasts for subcellular localization in wheat protoplasts by the PEG-mediated method (Yoo et al., 2007). After incubation at 25°C for 16 h, fluorescence signals were detected using a laser scanning confocal microscope (Leica TCS-NT, Germany). For observations of subcellular localization in tobacco leaf cells, constructs with the coding regions of TaPARG-2D and GFP as control, were transferred into tobacco leaves by *Agrobacterium*-mediated transformation (EHA105) (Liu et al., 2010), and cultured for 4 days at 22°C in a 16 h light/8 h darkness photoperiod.

### Analysis of Gene Expression

Total RNA was extracted with TRIzol, and cDNA was synthesized with a SuperScript^®^ Double-Stranded cDNA Synthesis Kit (Invitrogen Com., USA). Semi-quantitative RT-PCR was performed to analyze the expression pattern of *TaPARG* in various tissues. Primers P-TaPARG (5′- GGC CCTTCCACCCATATCA-3′ and 5′-GGTCACGCCACGGTA CATG-3′) and P-tubulin (5′-TGAGGACTGGTGCTTACCGC-3′ and 5′-GCACCATCAAACCTCAGGGA-3′) were used to amplify *TaPARGs* and the *tubulin* gene, respectively. Because *TaPARG-2A* and *TaPARG-2D* were highly similar in sequence, it was hard to design specific primers to separately amplify these two copies, therefore, the common primer P-TaPARG was used to amplify both *TaPARG-2A* and *TaPARG-2D* in wheat tissue expression analysis. The target PCR product size of P-TaPARG was 150 bp.

Different wheat tissues including roots, root bases, leaf sheaths, leaf blades, nodes, internodes, spikes, and plumules were collected to examine the expression pattern of *TaPARG* at various developmental stages (germination, seedlings, and heading).

### Functional Marker Development

Thirty-four accessions were initially chosen to detect nucleotide polymorphisms in *TaPARG-2A* coding and flanking regions. Three haplotypes were identified based on five SNPs (single nucleotide polymorphisms). Two specific functional markers (TaPARM1 and TaPARM2) were developed for the two SNPs in the exons. The primers of TaPARM1 were 5′-ACGCCACAGCCGAGAACGTCTTG-3′ and 5′-GG GGTGTACCTGGTGACGCCACAGT-3′, and the primers of TaPARM2 were 5′-GTTTTCGCACTGTCCCAAATC-3′ and 5′-GGACATGACGTCCTGGTTTC-3′. Genotyping was implemented by two rounds of PCR, the first was to amplify fragments from chromosome 2A in all cultivars with the genomic-specific primer pair F2/R2; the second was performed as follows: first round PCR products were diluted 100 times, and 1 μl of diluted solution was used as template for the second round using primers TaPARM1 and TaPARM2 (annealing at 55°C for 30 s, and extension at 72°C for 30 s). The PCR products were digested by the corresponding restriction enzymes and separated in 4% agarose gels.

### Population Structure and Association Analysis

Using data from 209 whole-genome SSR markers (Zhang et al., 2014), population structure was determined by STRUCTURE v2.3.4. Ten subpopulations (*k* = 1 to 10) were set with a burn-in period of 50,000 iterations and a run of 500,000 replications of Markov Chain Monte Carlo after burn in. The Δ*k* method was applied according to LnP (D) in the STRUCTURE, and the output and result were estimated (Li et al., 2015). The general linear model (GLM) in TASSEL v2.1 was used for association analysis, and *P* < 0.01 denotes significant association. Statistical analysis was conducted by SPSS v17.0, statistical significance was calculated using one-way ANOVA.

### Generation of Transgenic Plants in Rice

The cDNA of *TaPARG-2D* containing the entire ORF was inserted into pCUbi1390 vectors using primers 5′-GGGGTACC ATGACCAACAACAACGGCA-3′ (*Kpn*I site underlined) and 5′-CCCAAGCTTTCATGCGTCGCTCCACG-3′ (*Hin*dIII site underlined). The construct was transferred into rice. Positive plants were identified with hygromycin, and T3 generation transgenics overexpressing *TaPARG-2D* were reconfirmed by PCR.

## Results

### Cloning and Sequence Analysis of *TaPARG*

**FIGURE 1:**
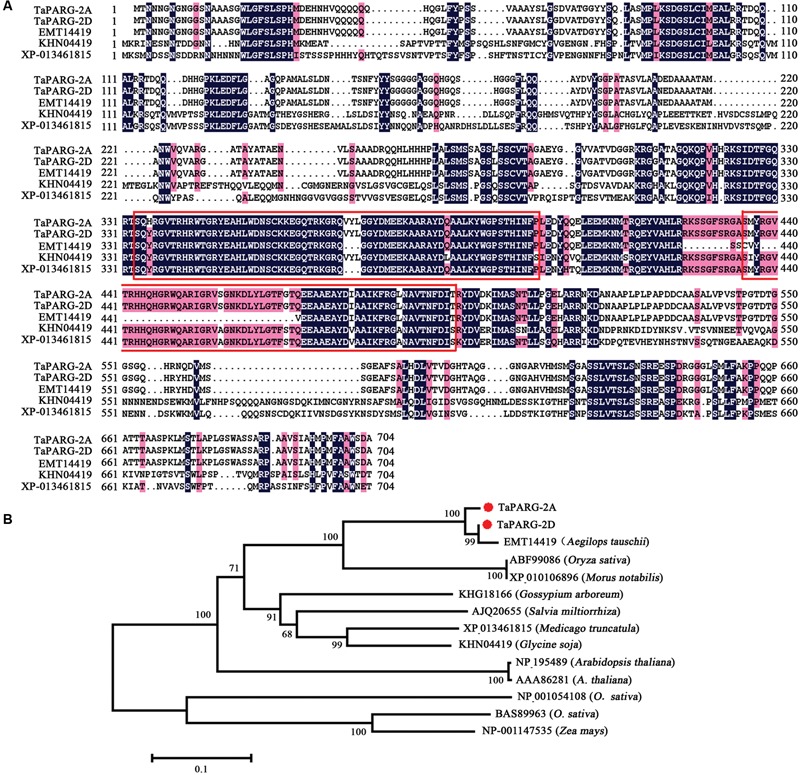
**Wheat TaPARGs belonging to the AP2/EREBP protein family. (A)** Alignment of AP2/EREBPs from different plant species; EMT14419 from *Aegilops tauschii*, KHN04419 from *Glycine soja*, and XP_-_013461815 from *Medicago truncatula*. Numbers indicate amino acid position. Common amino acid residues are shown in colored background. The conserved AP2 domains are marked in red rectangles. **(B)** Phylogenetic tree of AP2/EREBP proteins. The neighbor-joining tree was built with 2,000 bootstrap replicates. TaPARG-2A and TaPARG-2D are marked with red dots.

Two types of cDNA sequence of *TaPARG* were obtained from wheat cv. Hanxuan 10, and designated *TaPARG-2A* (GenBank AC: KU593503) and *TaPARG-2D* (GenBank AC: KU593504) according to their genomic origins. *TaPARG-2A* and *TaPARG-2D* have the same structure, containing eight exons and encoding proteins containing 601 and 600 amino acids, respectively. The identity of the two putative TaPARG proteins was 97.67%. The identities of TaPARG-2A with *Oryza sativa, Glycine soja*, and *Arabidopsis thaliana* were 53.10, 50.47, and 55.85%, respectively. Both TaPARGs contain two AP2 domains (**Figure 1A**). A neighbor-joining phylogenetic tree of AP2/EREBP2 was constructed for the full-length protein sequences of the TaPARGs with Clustal W (**Figure 1B**). The TaPARGs and the orthologous gene from *A. tauschii* were grouped into a separate clade. Protein identity and phylogeny analysis suggested that TaPARGs might have different functions compared to other AP2/EREBPs.

Since the two *TaPARGs* were highly similar in sequence, particularly in the AP2 domains, the D genomic member, *TaPARG-2D*, was chosen for functional analysis.

### Subcellular Localization of TaPARG-2D Protein

**FIGURE 2:**
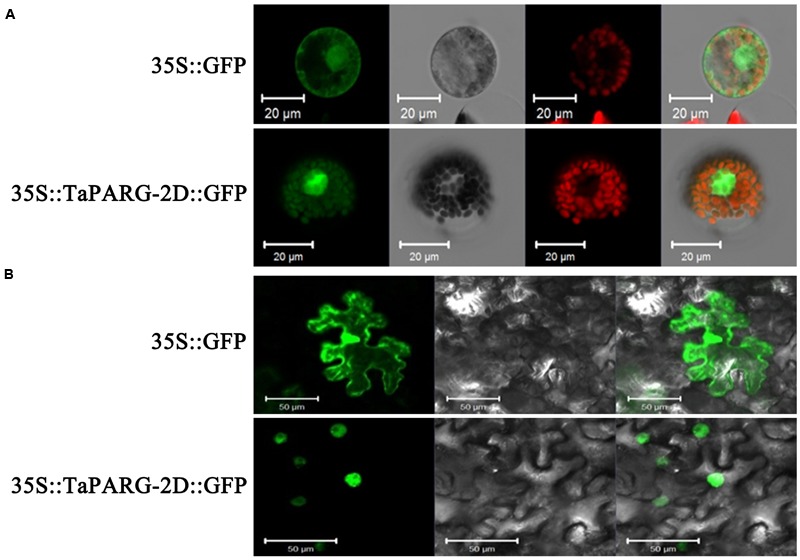
**Subcellular localization of TaPARG-2D in wheat protoplasts **(A)** and tobacco leaf cells **(B)**.** The vector control (35S::GFP) and fusion protein construct 35S::TaPARG-2D::GFP were introduced into wheat protoplasts and tobacco leaf cells, respectively. For wheat protoplast transformation, GFP was detected in transformed wheat lines after 24 h with a laser scanning confocal microscope; for tobacco, GFP was detected in leaf cells 4 days after transformation. Images are in dark field (1, 5, 9, and 12), bright field (2, 6, 10, and 13), and combined (4, 8, 11, and 14). Chloroplasts are indicated by red auto-fluorescence (3 and 7). Scale bars: 20 μm for wheat protoplasts; 50 μm for tobacco leaf cells.

AP2/EREBPs have roles as transcription factors (Shigyo et al., 2006). Prediction of subcellular localization using ProtComp Version 9.0 software suggested that TaPARG-2D was a typical nuclear localized protein. To further address this point, the recombinant construct of TaPARG-2D-GFP fusion protein was expressed in wheat protoplasts and tobacco leaves. TaPARG-2D was specifically located in the nucleus (**Figure 2**).

### Tissue Expression Pattern of *TaPARGs* in Wheat

**FIGURE 3:**
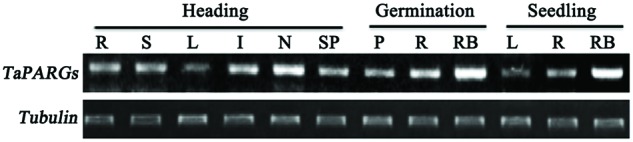
**Tissue expression patterns of *TaPARGs* in wheat.** Expression patterns at different developmental stages were detected by semi-quantitative RT-PCR. R, root; S, leaf sheath; L, leaf blade; I, internode; N, node; SP, spike; P, plumule; RB, root base.

Semi-quantitative RT-PCR was performed to analyze the tissue expression pattern of *TaPARGs* at different developmental stages in wheat (**Figure 3**). *TaPARGs* were constitutively expressed in different tissues at the germination, seedling, and heading stages, and were highly expressed in nodes and root bases. Constitutive expression suggested that *TaPARGs* might have active roles during the entire growth cycle.

### Overexpression of *TaPARG-2D* in Rice Influences Overall Plant Development

**FIGURE 4:**
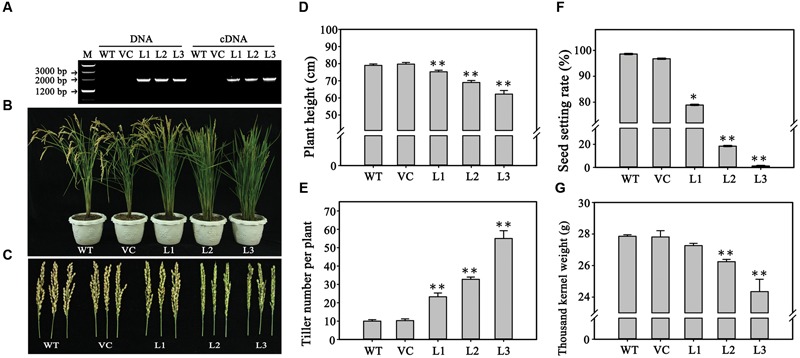
**Phenotypes of rice plants overexpressing *TaPARG-2D*. (A)** Gene amplification and transcriptional expression in three transgenic rice lines. Morphology of three transgenic lines and two controls: plants **(B)** and main panicles **(C)**. Comparison of agronomic traits in transgenic and control plants for plant height **(D)**, tiller number per plant **(E)**, seed setting rate **(F)** and 1,000 kernel weight **(G)**. WT, wild type; VC, plants transformed with the empty pCUBi1390 vector; L1, L2, and L3, transgenic lines. Error bars denote 1 SE. ^∗∗^*P* < 0.05, ^∗∗^*P* < 0.01, respectively.

Since *TaPARG* was constitutively expressed, we investigated the effects of overexpression in transformed rice plants harboring *TaPARG-2D* constructs driven by the CaMV 35S promoter. Eighteen transgenic lines were obtained. Three transgenic lines with different expression quantity were used for phenotype identification (**Figure 4A**; Supplementary Figure S1). Effects on plant growth (**Figure 4B**) and fertility (**Figure 4C**) were detected. Several biological and agronomic traits were measured to characterize the effects, including plant height (**Figure 4D**), tiller number per plant (**Figure 4E**), seed setting rate (**Figure 4F**), and 1,000 kernel weight (**Figure 4G**). With increasing relative expression levels of *TaPARG-2D* in different transgenic lines, plant height was reduced (from 75.2 to 62.3 cm) and tiller number per plant increased (from 23 to 55). Seed setting rate declined dramatically (from 78.9 to 1.3%) and 1,000 kernel weight decreased slightly (from 27.3 to 24.3 g). The overexpression lines exhibited significant changes in the growth and development of rice, which indicated *TaPARG-2D* performs functions in plant morphology formation.

### Genetic Mapping and Sequence Polymorphism

**FIGURE 5:**
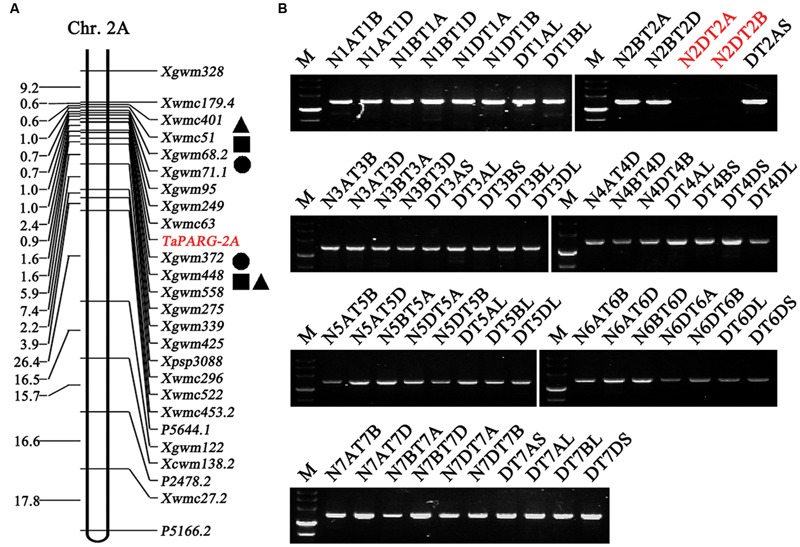
**Placement of *TaPARGs* on wheat chromosomes. (A)** *TaPARG-2A* was mapped on chromosome 2A flanked by *Xwmc63* and *Xgwm372*. Square, triangular, and circle spots indicate QTL for plant height, spike number per plant and grain weight, respectively. **(B)** *TaPARG-2D* was located on chromosome 2D using nulli-tetrasomic and ditelosomic lines of Chinese Spring. The target location and identified chromosome are in red. M, DNA Marker III (TransGen, Beijing).

Functional markers were used to scan the DH population derived from Hanxuan 10 × Lumai 14 (150 lines). *TaPARG-2A* was mapped to a region flanked by *Xwmc63* (0 cM) and *Xgwm372* (1 cM) on chromosome 2A (**Figure 5A**). *TaPARG-2D* was located on chromosome 2D using nulli-tetrasomic and ditelosomic lines of Chinese Spring (**Figure 5B**).

**FIGURE 6:**
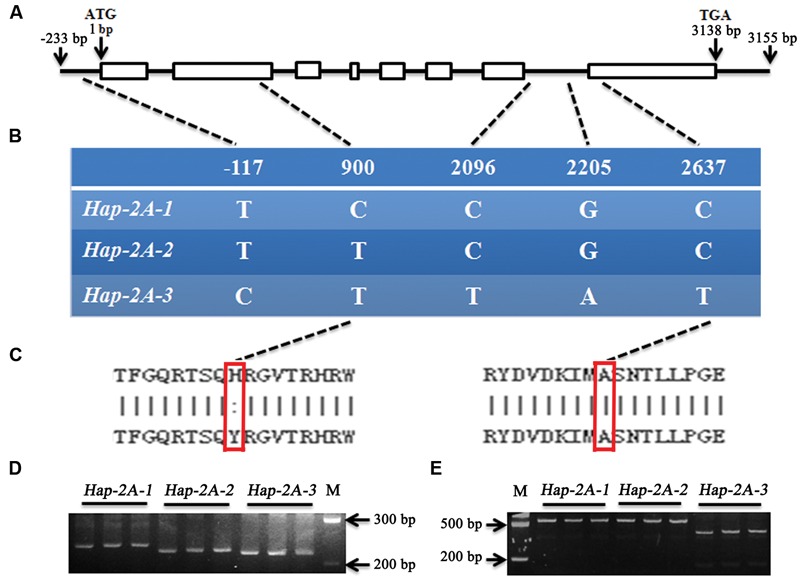
**Single nucleotide polymorphisms and functional markers of *TaPARG-2A*. (A)** Schematic diagram of *TaPARG-2A* structure. The ATG start codon is designated as position 1 bp. **(B)** Single nucleotide polymorphisms in three *TaPARG-2A* haplotypes identified among 34 wheat accessions. **(C)** Amino acid residues at SNP sites (red rectangles). **(D,E)** PCR products of functional markers TaPARM1 and TaPARM2 were restrictively digested by *Kpn* I and *Hin*d III, respectively. M, DNA Marker III (TransGen, Beijing).

The genomic sequences of *TaPARG-2A* and *TaPARG-2D* and flanking regions were obtained by searching the A and D genomic databases. Genome-specific primer pairs for the 2A (Primer F2/R2) and 2D (Primer F3/R3) genes were designed based on polymorphisms in the genome sequences. The *TaPARG-2A* and *TaPARG-2D* genomic fragments were 3,388 bp (**Figure 6A**) and 3,745 bp (Supplementary Figure S2), respectively. There was no nucleotide difference in the genomic region of *TaPARG-2D* among 34 accessions, but five variations (T/C, C/T, C/T, G/A, and C/T) were detected in the genomic region of *TaPARG-2A* (**Figure 6B**); three haplotypes were identified and named *Hap-2A-1, Hap-2A-2*, and *Hap-2A-3*. Only the SNP (C/T) in the second exon resulted in an amino acid change (His/Tyr; **Figure 6C**). Functional markers TaPARM1 and TaPARM2 were developed based on the SNPs at 900 bp (C/T) and 2637 bp (C/T), respectively. Amplified fragment length polymorphisms of TaPARM1 and TaPARM2 are shown in **Figures 6D,E**.

### Association Analysis of *TaPARG-2A* Haplotypes and Agronomic Traits

**FIGURE 7:**
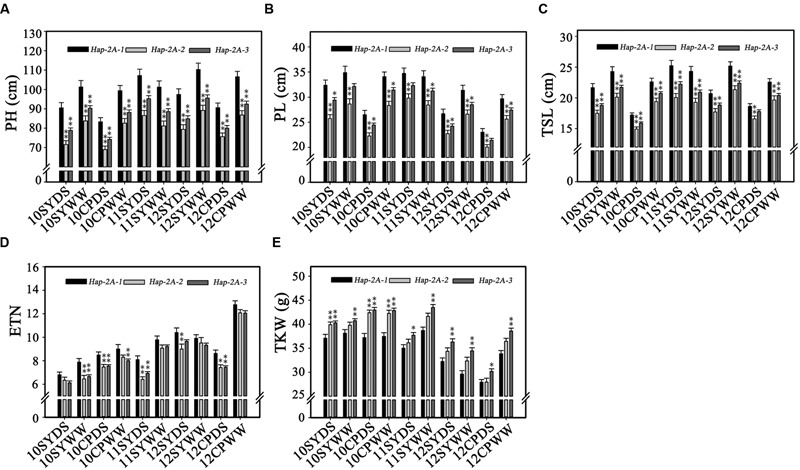
**Phenotypic comparisons of three *TaPARG-2*A haplotypes in 10 environments.** Traits are PH **(A)**, PL **(B)**, TSL **(C)**, ETN **(D),** and TKW **(E)**. ^∗^*P* < 0.05, ^∗∗^*P* < 0.01, respectively. Error bars denote 1 SE. See footnote to **Table 1** for abbreviations.

Population 1 (262 accessions) with an abundance of genetic diversity consisted of two sub-populations, comprising 126 and 136 accessions, based on genotyping results of 209 SSR markers (Zhang et al., 2014). Significant associations were identified between *TaPARG-2A* haplotypes and agronomic traits, including PH, PL, TSL, ETN, and TKW in all 10 environments (year × site × water regimes; **Table 1**). *Hap-2A-2* and *Hap-2A-3* were significantly associated with lower PH, PL, TSL and ENT, and higher TKW. However, there were no significant differences between genotypes possess *Hap-2A-2* and *Hap-2A-3* in any agronomic trait. The results were in good agreement in both DS and WW conditions (**Figure 7**). Thus, *Hap-2A-2* and *Hap-2A-3* might be favorable haplotypes for improvement of plant architecture and yield in wheat.

**Table 1:** *TaPARG-2A* haplotypes associations with agronomic traits in 10 environments.

Year	Site	Water regime	PH	PL	TSL	ETN	TKW
			*P*-value	*P*-value	*P*-value	*P*-value	*P*-value
2010	SY	DS	2.69E-08^∗∗∗^	1.10E-05^∗∗∗^	4.18E-07^∗∗∗^	n.s.	n.s.
	SY	WW	2.55E-05^∗∗∗^	6.94E-04^∗∗∗^	3.65E-05^∗∗∗^	0.0013^∗∗^	n.s.
	CP	DS	2.68E-07^∗∗∗^	5.50E-05^∗∗∗^	4.18E-07^∗∗∗^	3.73E-04^∗∗∗^	1.04E-07^∗∗∗^
	CP	WW	8.50E-07^∗∗∗^	3.49E-05^∗∗∗^	2.62E-05^∗∗∗^	0.0015^∗∗∗^	1.04E-07^∗∗∗^
2011	SY	DS	2.45E-05^∗∗∗^	0.0155^∗^	7.14E-05^∗∗∗^	1.29E-04^∗∗∗^	0.0106^∗^
	SY	WW	7.00E-07^∗∗∗^	1.33E-04^∗∗∗^	1.19E-06^∗∗∗^	0.0164^∗^	1.37E-05^∗∗∗^
2012	SY	DS	4.75E-05^∗∗∗^	0.0071^∗∗^	0.0044^∗∗∗^	0.0282^∗^	5.93E-04^∗∗∗^
	SY	WW	2.46E-06^∗∗∗^	0.0071^∗∗^	4.70E-04^∗∗∗^	n.s.	3.46E-05^∗∗∗^
	CP	DS	7.81E-05^∗∗∗^	n.s.	n.s.	1.44E-05^∗∗∗^	1.15E-06^∗∗∗^
	CP	WW	7.96E-07^∗∗∗^	0.0059^∗∗^	0.0035^∗∗^	n.s.	0.0238^∗^

*PH, plant height; PL, peduncle length; TSL, top 2nd internode length; ETN, effective tiller number; TKW, 1,000 kernel weight; n.s., not significant; ^∗^*P* < 0.05, ^∗∗^*P* < 0.01, and ^∗∗∗^*P* < 0.005, respectively. The 10 environments were at Changping (CP) and Shunyi (SY) under drought-stressed (DS) and well-watered (WW) conditions in 2010–2012.*

### The Effects of *TaPARG-2A* Haplotypes Were Confirmed in Population 3

**FIGURE 8:**
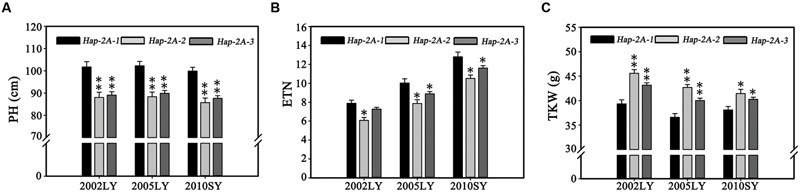
**Phenotypic comparison of *TaPARG-2A* haplotypes in the Population 3.** The population was grown in three environments for PH **(A)**, ETN **(B)**, and TKW **(C)** determinations. 2002LY, Luoyang 2002; 2005LY, Luoyang 2005; 2010SY, Shunyi 2010. ^∗^*P* < 0.05, ^∗∗^*P* < 0.01, respectively. Error bars denote 1 SE.

Population 3 (348 modern cultivars) accessions from the Chinese wheat core collection were used to further evaluate haplotype differences. Functional analysis of *TaPARG-2A* haplotypes in Population 3 (**Figure 8**; Supplementary Table S2) confirmed that *Hap-2A-2* and *Hap-2A-3* genotypes had lower PH and ETN, and higher TKW than *Hap-2A-1* genotypes; there were no significant differences between *Hap-2A-2* and *Hap-2A-3*, consistent with the previous results.

### Geographic Distribution of *TaPARG-2A* Haplotypes in Chinese Wheat Production Zones

**FIGURE 9:**
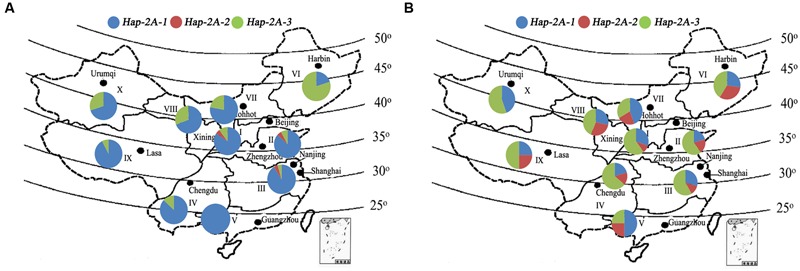
**Haplotype distribution of *TaPARG-2A* in 157 Chinese landraces (Population 2; **A)** and 348 modern cultivars (Population 3; **B)** in 10 major wheat zones.** I, Northern Winter Wheat Zone; II, Yellow and Huai River Valleys Facultative Wheat Zone; III, Middle and Low Yangtze Valleys Autumn-Sown Spring Wheat Zone; IV, Southwestern Autumn-Sown Spring Wheat Zone; V, Southern Autumn-Sown Spring Wheat Zone; VI, Northeastern Spring Wheat Zone; VII, Northern Spring Wheat Zone; VIII, Northwestern Spring Wheat Zone; IX, Qinghai-Tibetan Plateau Spring-Winter Wheat Zone; X, Xinjiang Winter-Spring Wheat Zone. The maps were generated using Mapinfo Professional 11.0 software.

Common wheat in China is grouped into three types according to time of sowing and vernalization requirement, namely, spring, facultative, and winter types. The national wheat production area is divided into 10 major agro-ecological production zones based on ecological conditions, variety type and cropping season (Jin, 1983). Accessions in Population 2 (157 landraces) and Population 3 (348 modern cultivars) covering all 10 zones were used to evaluate the geographic distributions of *TaPARG-2A* haplotypes. *Hap-2A-1* is the dominant haplotype in landraces in all zones, except Zone VI, i.e., the Northeastern Spring Wheat Zone, where *Hap-2A-3* is the dominant haplotype (**Figure 9A**). However, among modern cultivars, the combined frequency of the two favored haplotypes (*Hap-2A-2* and *Hap-2A-3*) were higher in Zones I–IV (70–80%) than in Zones V–X (50–75%; **Figure 9B**). The zones with higher proportions of favored haplotypes are the major production regions, where the frequency of release of new varieties is higher than other regions.

### Favored Haplotypes of *TaPARG-2A* Were Selected by Wheat Breeders

**FIGURE 10:**
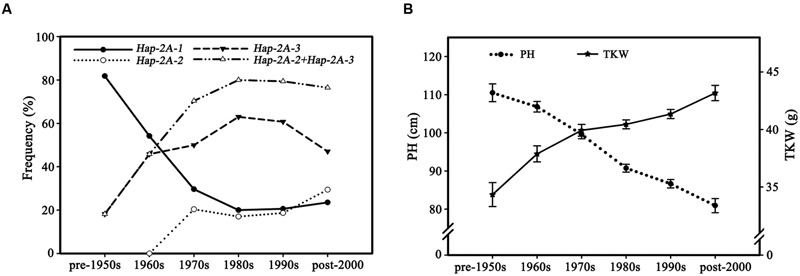
**Favored haplotypes were selected in Chinese wheat breeding programs. (A)** *TaPARG-2A* haplotype frequencies in 335 modern cultivars (Population 3) over decades. 12, 25, 55, 102, 106, and 35 accessions were released in the pre-1950s, 1960s, 1970s, 1980s, 1990s, and post-2000, respectively. **(B)** PH and TKW changes in 335 modern cultivars (Population 3) over decades. Error bars denote 1 SE.

To assess changes in haplotype frequency over time 335 accessions in Population 3 with known dates of release were divided into six subgroups according to decade of release (Pre-1950s, 1960s, 1970s, 1980s, 1990s, and Post-2000). The proportion of *Hap-2A-1* genotypes decreased significantly, from 81.8% in the pre-1950s cohort to 20.6% in the 1980s, correspondingly the frequencies of *Hap-2A-2* and *Hap-2A-3* increased from 18.2 to 79.4% (**Figure 10A**), and the PH also significantly reduced and TKW significantly increased (**Figure 10B**). These data strongly suggest that the two favored haplotypes, *Hap-2A-2* and *Hap-2A-3*, were apparently selected in Chinese wheat breeding programs before the 1980s. The proportions of the favored haplotypes have maintained a relatively high and stable level since the 1980s, but the PH and TKW continued earlier trends implying that other genes affecting these traits were selected after the 1980s.

## Discussion

Genes *TaPARG-2A* and *TaPARG-2D*, containing two highly conserved AP2 domains, belong to the AP2 subfamily of AP2/EREBP transcription factors, that are involved in regulation of diverse processes of plant development and stress response. Both *AP2* and *ANT* have important roles in reproductive organ development in angiosperm species (Shigyo and Ito, 2004). A series of AP2-like transcription factors have been characterized. *AINTEGUMENTA-like6* (*AIL6*) is an important regulator of floral growth and patterning (Krizek, 2009). Another AP2-like gene *Indeterminate spikelet 1* (*IDS1*), suppresses indeterminate growth within the spikelet meristem (Chuck et al., 1998). The functions of a gene family are generally conserved, but in some cases functions may be divergent. For example, TaPARGs, members of the AP2/EREBP transcription factor family, differ from other proteins in the family and are therefore classified into a separate branch with the *A. tauschii* protein. *TaPARG-2A* was mapped to a region flanked by *Xwmc63* and *Xgwm372* on chromosome 2A, adjacent to QTL for several agronomic traits (**Figure 5**) including PH (Wu et al., 2010), spike number per plant (Wu et al., 2011, 2012) and grain weight (Li et al., 2012). Two conditional QTLs affecting PH were separately flanked by *Xwmc51*-*Xgwm68.2* and *Xgwm448*-*Xgwm558*, and the genetic distances between QTLs for PH and *TaPARG-2A* were 2.4 and 1.0 cM, respectively. The nearest markers of QTLs for spike number per plant were *Xwmc401* and *Xgwm448*, and the genetic distances between QTLs for spike number per plant and *TaPARG-2A* were 4.0 and 1.0 cM, respectively. Two QTLs for grain weight were flanked by *Xgwm68.2*-*Xgwm71.7* and *Xgwm372*-*Xgwm448*, and the genetic distances between the QTLs and *TaPARG-2A* were 1.7 and 1.0 cM, respectively. *TaPARG-2A* and three QTLs flanked by *Xgwm372*-*Xgwm558* were located in the centromere region of chromosome 2A^1^, controlling the traits of grain weight, plant height, and spike number per plant, respectively, and all of them with 1.0 cM away from *TaPARG-2A*. By the method proposed by Visscher and Goddard (2004), *TaPARG-2A* fell into the 95% confidence intervals of all of these three QTLs. Gene expression studies showed that *TaPARGs* were expressed in multiple tissues at three growth stages, suggesting roles in multiple developmental processes as a pleiotropic gene. Overexpression of *TaPARG-2D* in rice caused a range of changes, including reduced plant height, seed setting rate and 1,000 kernel weight, but an increase in tiller number per plant. Association analysis of different germplasm populations indicated that *TaPARG-2A* affects several agronomic traits (PH, PL, TSL, ETN, and TKW). All these data show that *TaPARGs* are novel, pleiotropic genes in the AP2/EREBP gene family, and are involved in regulation of plant growth and development. The present research suggests that *TaPARG* is a regulatory factor in plant growth and development by rice transgenic lines and association analysis in wheat. In the future, the gene expression and function should be further verified in same plant species to minimize the influence of genetic backgrounds.

Wheat was domesticated about 10,000 years ago (Gill et al., 2004). In two rounds of polyploidization, common wheat evolved with only part of the genetic diversity of its progenitors and subsequently generated new diversity by mutation tolerated by genetic duplication and redundancy (Dubcovsky and Dvorak, 2007). We identified *TaPARG-2D* was highly conserved and involved in regulating several agronomic traits, including plant height, tiller number per plant, seed setting rate, and 1,000 kernel weight. Three haplotypes of *TaPARG-2A* in hexaploid wheat based on five SNPs. According to an association analysis of *TaPARG-2A* genotype and agronomic traits, the three haplotypes were divided into favored (*Hap-2A-2* and *Hap-2A-3*) and unfavored (*Hap-2A-1*) haplotypes for wheat improvement.

**FIGURE 11:**
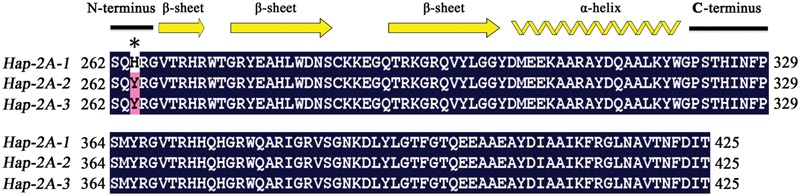
**Comparison of AP2 domain amino acid sequences in three haplotypes of *TaPARG-2A*.** Secondary structural units are shown at the top. Common amino acid residues are shown in black background. Numbers indicate amino acid position. Asterisk indicates variation site.

The amino acid residues (Arg147 and Gly148) in the GBD have backbone-binding function (Allen et al., 1998), and the equivalent functional loci in *TaPARG-2A* were Arg265 and Gly266. Five SNPs in *TaDGRG-2A* were identified among the accessions examined, but only one nucleotide difference (C/T) at 900 bp led to an amino acid change, i.e., His264 to Tyr264 (**Figure 11**). Histidine is an alkaline amino acid, whereas Tyrosine is a polar neutral amino acid. As the SNP change was closely adjacent to the DNA binding sites Arg265 and Gly266, the change likely influences interaction between TaPARG-2A and its target *cis*-element, and thus could result in a functional difference. The same single amino acid difference was present in two agronomically favored *TaPARG-2A* haploytpes, *Hap-2A-2* and *Hap-2A-3*.

Wheat domestication is a process of accumulating superior genotypes and phenotypes. Increasing applications of nitrogenous fertilizer in traditional wheat management did not initially increase grain yield because the crop became too tall and lodged. The adoption of lodging-resistant wheat permitted higher cereal crop yields from the 1960s, and the “Green Revolution” was fueled in part by the introduction of semidwarf wheat varieties carrying *Rht* mutations (Borner et al., 1996). Almost all current commercial wheat varieties carry one of the *Rht* mutant alleles (Silverstone and Sun, 2000). Thus rational development and utilization of height-reducing genes made a huge contribution to increasing wheat production. Our research showed that the favored *Hap-2A-2* and *Hap-2A-3* haplotypes of *TaPARG-2A* were selected in the process of wheat improvement in China. From landraces to recent modern cultivars, the proportion of the favored haplotypes increased in stepwise manner. This increase was more apparent in the major wheat-production regions where there was a greater turnover of cultivars. The frequencies of the two favored haplotypes rose steadily from the 1950 to 1980s, during which time Chinese wheat varieties underwent very significant reductions in plant height and increases in grain yield. Given that the favored haplotypes of *TaPARG-2A* were apparently selected in the Chinese wheat breeding history, further selection of favored alleles might be useful for continued improvement of plant architecture and grain yield.

Plant breeding through phenotypic selection is a time-consuming and relatively inefficient process (Gedye et al., 2012). Fortunately, the wheat community has made remarkable progress in developing molecular resources for breeding (Paux et al., 2012). Functional markers also called perfect or diagnostic markers have been developed for many traits including plant architecture, disease resistance, and grain quality (Gedye et al., 2012). In this study, after functional verification of *TaPARG*, a functional marker (TaPARM1) was developed to identify the favored *Hap-2A-2* and *Hap-2A-3* haplotypes. Currently, the combined frequency of the two favored haplotypes is about 80%. Utilization of the functional marker should contribute to further improvement of plant architecture and grain yield in wheat.

## Author Contributions

Conceived and designed the experiments: RJ and BL. Performed the experiments: BL, QL, XM, AL, JW, XC, XZ, and CH. Analyzed the date: BL. Contributed to the writing of the manuscript: BL and RJ. Revised the manuscript: BL and RJ.

## Conflict of Interest Statement

The authors declare that the research was conducted in the absence of any commercial or financial relationships that could be construed as a potential conflict of interest.

## Footnotes

1 http://wheat.pw.usda.gov/cgi-bin/GG3/report.cgi?class=image;name=Ta-Synthetic%2FOpata-SSR+group+2+maps
