# Supplementary material for: Two Novel AP2/EREBP Transcription Factor Genes TaPARG Have Pleiotropic Functions on Plant Architecture and Yield-Related Traits in Common Wheat
Source: Front Plant Sci. 2016 Aug 9;7:1191. doi: 10.3389/fpls.2016.01191 (PMC4977303; doi:10.3389/fpls.2016.01191)
Supplement: Supplementary file 1 [file Table_1.PDF]

Title

Two novel AP2/EREBP transcription factor gene *TaPARG* has pleiotropic functions on plant architecture and yield-related traits in common wheat

Authors and addresses

First author: Bo Li, Qiaoru Li

Corresponding author: Ruilian Jing

Order of Authors: Bo Li*, Qiaoru Li*, Xinguo Mao, Ang Li, Jingyi Wang, Xiaoping Chang, Chenyang Hao, Xueyong Zhang & Ruilian Jing

National Key Facility for Crop Gene Resources and Genetic Improvement/Institute of Crop Science, Chinese Academy of Agricultural Sciences, Beijing 100081, China.

*These authors contributed equally to this work.

Correspondence:

National Key Facility for Crop Gene Resources and Genetic Improvement/Institute of Crop Science, Chinese Academy of Agricultural Sciences, Beijing 100081, China.

Tel/Fax: +86 (0)10 82105829

E-mail: jingruilian@caas.cn

Supplementary Information

Supplementary Fig. S1. Relative expression levels of *TaPARG-2D* in three transgenic rice lines.

Supplementary Fig. S2. Schematic diagram of *TaPARG-2D* gene structure.

Supplementary Table S1. Cultivars used for haplotype identification.

Supplementary Table S2. Trait phenotypes in Population 3 of *TaPARG-2A* haplotypes grown in three environments.

Supplementary Table S1. Cultivars used for haplotype identification.

Cultivar	Origin	Time of release
Beijing 10	Beijing	1960s
Hanxuan 10	Shanxi	1960s
An 85 Zhong 124-1	Beijing	1970s
Dan R 8093	Beijing	1970s
Jingpin 10	Beijing	1970s
Jimai 6	Hebei	1970s
Changle 5	Shandong	1970s
Dali 1	Shaanxi	1970s
Beijing 14	Beijing	1980s
Fengkang 13	Beijing	1980s
Jinghe 8922	Beijing	1980s
Jin 2148-7	Fujian	1980s
Changwu 131	Shaanxi	1980s
04-030	Beijing	1990s
04-044	Beijing	1990s
Beijing 8686	Beijing	1990s
Jing 411	Beijing	1990s
Jimai 41	Hebei	1990s
Neixiang 188	Henan	1990s
Yanzhan 1	Henan	1990s
Pandas	Italy	1990s
Lumai 14	Shandong	1990s
Chun 9th-25	CIMMITY, Mexico	2000s
Chun 9th-5-1	CIMMITY, Mexico	2000s
Chun 9th-50-1	CIMMITY, Mexico	2000s
Chang 6878	Shanxi	2000s
Liangxing 99	Shandong	2000s
Linkang 5108	Shanxi	2000s
Baiqimai	Gansu	Landrace
Baicaomai	Henan	Landrace
Ziganbaimangxian	Henan	Landrace
Cangzhouxiaomai	Hebei	Landrace
Hongheshang	Shanxi	Landrace
Chinese Spring	Sichuan	Landrace
