# Supplementary material for: Two Novel AP2/EREBP Transcription Factor Genes TaPARG Have Pleiotropic Functions on Plant Architecture and Yield-Related Traits in Common Wheat
Source: Front Plant Sci. 2016 Aug 9;7:1191. doi: 10.3389/fpls.2016.01191 (PMC4977303; doi:10.3389/fpls.2016.01191)
Supplement: Supplementary file 2 [file Table_2.PDF]

Title

Two novel AP2/EREBP transcription factor gene *TaPARG* has pleiotropic functions on plant architecture and yield-related traits in common wheat

Authors and addresses

First author: Bo Li, Qiaoru Li

Corresponding author: Ruilian Jing

Order of Authors: Bo Li[*], Qiaoru Li[*], Xinguo Mao, Ang Li, Jingyi Wang, Xiaoping Chang, Chenyang Hao, Xueyong Zhang & Ruilian Jing

National Key Facility for Crop Gene Resources and Genetic Improvement/Institute of Crop Science, Chinese Academy of Agricultural Sciences, Beijing 100081, China.

[*]These authors contributed equally to this work.

Correspondence:

National Key Facility for Crop Gene Resources and Genetic Improvement/Institute of Crop Science, Chinese Academy of Agricultural Sciences, Beijing 100081, China.

Tel/Fax: +86 (0)10 82105829

E-mail: jingruilian@caas.cn

Supplementary Information

Supplementary Fig. S1. Relative expression levels of *TaPARG-2D* in three transgenic rice lines.

Supplementary Fig. S2. Schematic diagram of *TaPARG-2D* gene structure.

Supplementary Table S1. Cultivars used for haplotype identification.

Supplementary Table S2. Trait phenotypes in Population 3 of *TaPARG-2A* haplotypes grown in three environments.

Supplementary Table S2. Trait phenotypes in Population 3 of *TaPARG-2A* haplotypes grown in three environments.

Year	Site	Trait	Haplotype		
			Hap-2A-1	***Hap-2A-2***	***Hap-2A-3***
2002	LY	PH	101.7±2.4A	88.0±2.4B	89.1±1.5B
	LY	ETN	7.9±0.3a	6.1±0.3b	7.3±0.2a
	LY	TKW	39.4±0.8A	45.6±0.8B	43.2±0.5B
2005	LY	PH	102.3±1.9A	88.4±2.1B	89.8±1.3B
	LY	ETN	10.0±0.5a	7.9±0.4b	8.9±0.2b
	LY	TKW	36.6±0.76A	42.7±0.61B	40.0±0.5B
2010	SY	PH	99.9±1.7A	85.7±2.1B	87.7±1.2B
	SY	ETN	12.8±0.5a	10.5±0.4b	11.6±0.2b
	SY	TKW	38.1±0.7a	41.5±0.9b	40.3±0.4b

Population 3 was planted at Luoyang (LY) and Shunyi (SY) in 2002, 2005 and 2010. Lower and upper case letters in columns indicate significant differences at $P < 0.05$ and 0.01, respectively.
