# Supplementary figures and images for: Two Novel AP2/EREBP Transcription Factor Genes TaPARG Have Pleiotropic Functions on Plant Architecture and Yield-Related Traits in Common Wheat
Source: Front Plant Sci. 2016 Aug 9;7:1191. doi: 10.3389/fpls.2016.01191 (PMC4977303; doi:10.3389/fpls.2016.01191)

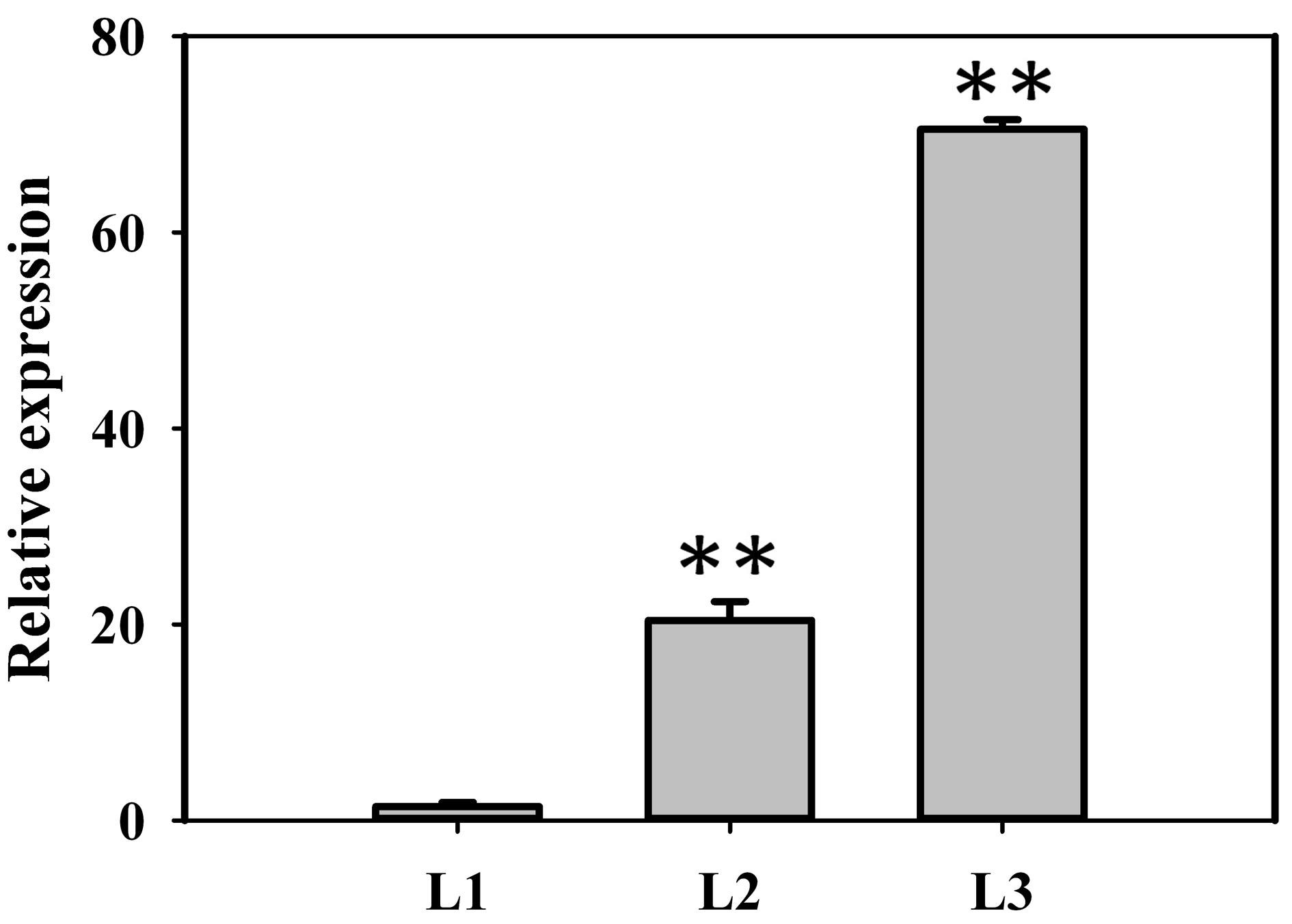

Supplement: Supplementary file 3 [file Image_1.TIF]

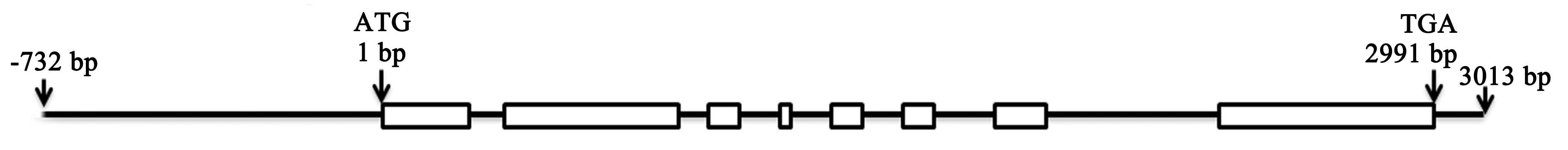

Supplement: Supplementary file 4 [file Image_2.TIF]
